# Citrullination Licenses Calpain to Decondense Nuclei in Neutrophil Extracellular Trap Formation

**DOI:** 10.3389/fimmu.2019.02481

**Published:** 2019-10-22

**Authors:** Stefanie Gößwein, Aylin Lindemann, Aparna Mahajan, Christian Maueröder, Eva Martini, Jay Patankar, Georg Schett, Christoph Becker, Stefan Wirtz, Nora Naumann-Bartsch, Marco E. Bianchi, Peter A. Greer, Günter Lochnit, Martin Herrmann, Markus F. Neurath, Moritz Leppkes

**Affiliations:** ^1^Department of Medicine 1, Friedrich-Alexander-Universität Erlangen-Nürnberg, Deutsches Zentrum Immuntherapie, Kussmaul Campus for Medical Research and Translational Research Center, Erlangen, Germany; ^2^Department of Medicine 3—Rheumatology and Immunology, Friedrich-Alexander-University Erlangen-Nürnberg and Universitätsklinikum Erlangen, Erlangen, Germany; ^3^Department of Pediatrics, Friedrich-Alexander-University Erlangen-Nürnberg and Universitätsklinikum Erlangen, Erlangen, Germany; ^4^Chromatin Dynamics Unit, Division of Genetics and Cell Biology, IRCCS San Raffaele Scientific Institute, Milan, Italy; ^5^Department of Pathology and Molecular Medicine, Queen's University, Kingston, ON, Canada; ^6^Institute of Biochemistry, Justus-Liebig-Universität Gießen, Giessen, Germany

**Keywords:** neutrophil, citrullination, PAD4, calpain, cell death, nucleus, chromatin

## Abstract

Neutrophils respond to various stimuli by decondensing and releasing nuclear chromatin characterized by citrullinated histones as neutrophil extracellular traps (NETs). This achieves pathogen immobilization or initiation of thrombosis, yet the molecular mechanisms of NET formation remain elusive. Peptidyl arginine deiminase-4 (PAD4) achieves protein citrullination and has been intricately linked to NET formation. Here we show that citrullination represents a major regulator of proteolysis in the course of NET formation. Elevated cytosolic calcium levels trigger both peptidylarginine deiminase-4 (PAD4) and calpain activity in neutrophils resulting in nuclear decondensation typical of NETs. Interestingly, PAD4 relies on proteolysis by calpain to achieve efficient nuclear lamina breakdown and chromatin decondensation. Pharmacological or genetic inhibition of PAD4 and calpain strongly inhibit chromatin decondensation of human and murine neutrophils in response to calcium ionophores as well as the proteolysis of nuclear proteins like lamin B1 and high mobility group box protein 1 (HMGB1). Taken together, the concerted action of PAD4 and calpain induces nuclear decondensation in the course of calcium-mediated NET formation.

## Introduction

In recent years, a functional role of neutrophil extracellular traps (NETs) has been implicated in the setting of multiple diseases as diverse as autoimmunity, cancer, thrombosis, crystalopathies, and the response to large pathogens (1, 2). NETs serve an important role in the containment and remodeling of inflammatory foci. Chromatin decondensation is a crucial event in the formation of NETs (3). In prototypical apoptosis, nuclear chromatin condensation and caspase-dependent DNase activity result in characteristic DNA fragmentation at the internucleosomal level (4). On the other hand, NETs capable of pathogen immobilization and thrombus stabilization depend on large undegraded tangles of chromatin, which functionally depend on the framework of extracellular DNA (5, 6). The mechanism of chromatin decondensation in the setting of NET formation is incompletely understood. Proteolytic cleavage events by neutrophil serine proteases such as neutrophil elastase have been implicated in neutrophils treated with phorbol esters acting in conjunction with reactive oxygen species (ROS) (7). Pharmacological inhibitors of peptidylarginine deiminases (PAD) and mice deficient in PAD4 (the only PAD enzyme with a nuclear localization signal) have been used in a wide variety of experiments to model deficient NET formation (8–10). PAD activity induces the modification of peptide-associated basic arginine residues to neutral citrulline. Citrullination thus disturbs polar protein interactions and the modification of the histone N-terminus is thought to diminish histone-DNA binding and account for chromatin decondensation. However, the role of citrullination by PADs as one of a multitude of potential posttranslational histone modifications remains enigmatic (11–14): In the setting of multiple posttranslational chromatin modifications *in vivo*, can loss of charge in arginine residues of histone proteins alone account for the massive decondensation observed in NET formation? Why should chromatin decondensation be sufficient to decondense an entire nucleus featuring a proteinaceous nuclear cytoskeleton and nuclear lamina? Currently, protein citrullination by PAD4 is not sufficiently integrated into previously defined signaling pathways of cellular demise and its function remains poorly defined. Its role in PMA- or pathogen-evoked NET formation has even been questioned recently (11, 15), while convincing results link PAD4-mediated NET formation to thrombus maturation (10), calling for a reevaluation of its role in NET formation.

Programmed cell death is typically associated with elevated protease activity. Apoptosis is characteristically driven by initiator and effector caspases (16). Caspase activity may be induced by extrinsic or intrinsic mechanisms. Additional protease cascades exist, which determine regulated necrotic cell death independently of caspase activity, often lacking characteristic morphological features of caspase-mediated apoptosis (17). Many years ago, members of the calpain family were identified as important calcium-dependent proteases in necrotic cell death (18). Calpains feature a peculiar mode of proteolytic cleavage. As opposed to tryptic-type serine proteases, which specifically cleave after arginine and lysine residues, calpain-mediated proteolysis is guided by the three-dimensional architecture of the substrate proteins and relies on the specific conformation of the protein (19): Large cytoskeletal proteins are hallmark substrates of calpain. Here, a regulated cleavage by calpain is achieved by controlled cleavage between domains, as observed for spectrin repeat domain proteins.

In this study, we observed that independent pathways lead to chromatin decondensation in neutrophils, typical of NETs. Moreover, these independent NET-forming pathways are characterized by differential proteolysis of nuclear membrane and chromatin proteins, and PAD4 intricately regulates substrate vulnerability to proteolysis (20). Citrullination by PAD4 is not necessary for elastase-mediated chromatin decondensation. However, PAD4 strongly enhances the capability of calpain to induce chromatin decondensation. Calpain alone did not induce chromatin decondensation in the absence of PAD4, whereas the concerted action of both was able to strongly decondense nuclear chromatin. We identified multiple proteins as relevant targets in this process including but not limited to high-mobility group box 1 protein (HMGB1), histone H3, lamins, and HP1 chromobox proteins.

## Results

### Independent Pathways Induce Chromatin Decondensation in Neutrophils

**Figure 1:**
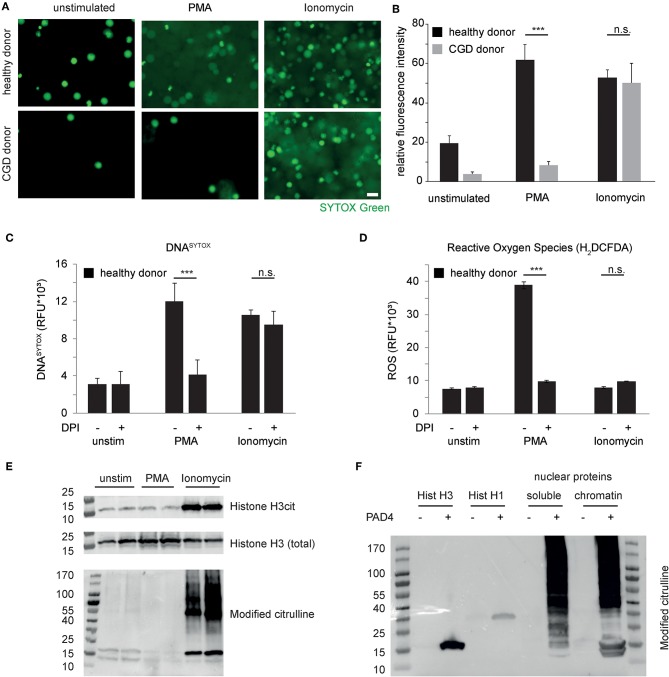
Independent pathways induce chromatin decondensation in neutrophils. **(A)** Isolated neutrophils were stimulated in HBSS media with phorbol myristyl acetate (PMA, 50 nM) or ionomycin (5 μM). Imaging was performed 3 h after stimulation. The reproducibly observed response of peripheral blood neutrophils derived from healthy donors and a patient suffering from chronic granulomatous disease (CGD) is depicted. DNA of neutrophils is stained by SYTOX Green (in green), once the plasma membrane is disrupted and/or DNA has been expulsed. Decondensation of chromatin results in increased area and decreased intensity of SYTOX staining (scale bar = 20 μm). **(B)** SYTOX Green fluorescence intensity of microscopic images from equal numbers of cells as presented in **(A)** was quantified and represented as relative signal intensity (^***^p < 0.001 Student's *t*-test of technical replicates, *n* > 4 healthy donors, *n* = 2 independent experiments with CGD neutrophils). **(C,D)** Stimulation of healthy donor-derived human neutrophils in the presence or absence of Diphenyliodonium (DPI) and the previously mentioned stimuli and **(C)** whole-well DNA^SYTOX^ quantification as well as **(D)** whole well detection of reactive oxygen species (H2-DCFDA) was performed after 180 min in a Tecan M200 microplate reader. Results representative of 4 independent healthy donors. **(E)** Neutrophil granulocytes were stimulated with the indicated stimuli for 120 min before subsequent protein isolations. Citrullinated Histone H3, total histone H3 and citrulline-containing proteins were assessed subsequently. **(F)** Recombinant Histone H3 and Histone H1 as well as nuclear protein fractions of A549 cells were treated with recombinant PAD4. Protein citrullination was assessed by antibody based detection of modified citrulline residues after reaction with diacetyl monoxime/antipyrine. Unless otherwise indicated, results are representative of at least 3 independent experiments using independent healthy donors.

To study the molecular mechanisms of NET formation, neutrophils were stimulated with prototypical instigators of NET formation, namely phorbol-myristate-acetate (PMA), known to result in protein kinase C activation, the assembly of the NAPDH oxidase complex and instigation of the oxidative burst. Additionally, neutrophils were stimulated with the bacteria-derived calcium ionophore ionomycin. Analyses by fluorescence microscopy revealed striking chromatin decondensation in response to both stimuli in healthy donor-derived neutrophils, whereas chromatin decondensation in response to PMA was drastically reduced in neutrophils derived from a patient with chronic granulomatous disease (CGD) (Figures 1A,B). However, ionomycin was perfectly capable of inducing chromatin decondensation even in the absence of the respiratory burst in CGD patient-derived neutrophils. Pharmacological inhibition of the NADPH oxidase complex by means of diphenyliodonium (DPI) revealed compatible findings (Figure 1C). DPI failed to inhibit ionomycin-induced chromatin externalization as detected using whole-well detection of DNA^SYTOX^, whereas DPI was capable of inhibiting PMA-induced chromatin externalization. PMA induced a marked ROS generation in healthy neutrophils, whereas ionomycin did not (Figure 1D). As observed previously (21), these results point to a divergent necessity of NADPH-oxidase-derived ROS for independent pathways of chromatin decondensation in human neutrophils.

Recent publications have highlighted the role of peptidyl arginine deiminases and protein citrullination as a mechanistic feature of NET formation (9, 12, 13). Independent groups have shown citrullinated histone H3 as a hallmark feature of NETs (9, 22). In the prevailing view, citrullination of histone H3 causes a loss of charge of basic chromatin-bound proteins and thereby results in massive chromatin decondensation (12). We studied citrullination events in the course of both PMA- and ionomycin-induced NET formation *in vitro*. These experiments showed that, 120 minutes after stimulation, marked citrullination of histone H3 is restricted to calcium-ionophore treated neutrophils as opposed to untreated and PMA-treated cells under the specified experimental conditions (Figure 1E). Interestingly, citrullination is not restricted to histone H3 but rather affects a large number of proteins of various molecular sizes as assessed by specific immunodetection of modified citrulline residues. Processing by recombinant PAD4 of histone proteins and isolated nuclear protein fractions again showed low substrate specificity and rather broad protein citrullination by PAD4 (Figure 1F) (23). Taken together, neutrophil chromatin decondensation can be achieved via independent pathways (11). On the one hand, a need for NADPH-oxidase derived ROS is indicated. On the other hand, calcium ionophore-treated neutrophils are characterized by marked protein citrullination, which is not restricted to histones.

### Differential Proteolytic Cleavage Events in NET Formation

**Figure 2:**
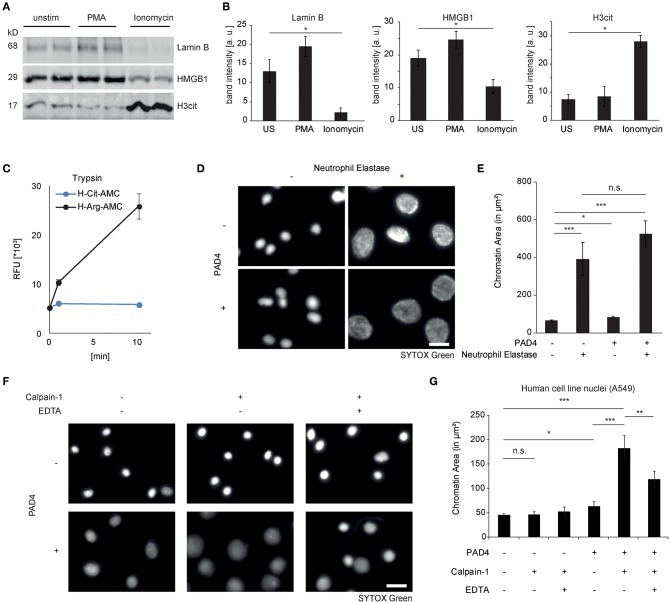
Differential proteolytic cleavage events in NET formation. **(A**) Lamin B1, HMGB1, and citrullinated Histone H3 were assessed by western blotting in lysates from human neutrophils after stimulation for 120 min with PMA or ionomycin (*n* = 3 independent healthy donors). **(B)** Band intensity calculations of western blots as in **(A)** were performed using Image J-assisted density quantification of at least 4 lanes (^**^p < 0.01 ANOVA and *post-hoc* Tukey HSD test). **(C)** The fluorogenic substrates H-Cit-AMC (25 μM) and H-Arg-AMC (25 μM) were subjected to trypsin digestion and emitted fluorescence was measured. **(D)** Isolated A549 nuclei were subjected to neutrophil elastase (60nM) for 4 h after preincubation in the presence or absence of PAD4 (5 μM) for 90 min (scale bar = 20 μm). **(E)** Area of SYTOX Green-stained single nuclear chromatin was quantified (at least 100 nuclei in each of 3 independent experiments, depicted as mean + standard error, ^*^p < 0.05, ^***^p < 0.001, ANOVA and *post-hoc* Tukey HSD test). **(F)** Isolated A549 nuclei were subjected to human calpain-1 for 4 h after preincubation in the presence or absence of PAD4 (5 μM) for 60 min (scale bar = 20 μm). **(G)** Nuclear chromatin area of SYTOX-Green-stained single nuclei was quantified as described in **(E)**.

Programmed cell death pathways are characterized by proteolysis of intracellular target proteins (24). As loss of nuclear integrity is a characterizing feature of NET formation, we studied proteolysis of nuclear proteins in healthy donor-derived neutrophils after PMA or ionomycin challenge. Interestingly, ionomycin induced a marked loss of the nuclear envelope protein Lamin B1 as well as of the nuclear chromatin-binding protein high mobility group box protein 1 (HMGB1) in the course of 2 hours, which was not yet observed after 120 minutes of PMA stimulation (Figures 2A,B). As citrullination is a key feature of ionophore-stimulated neutrophils, we asked whether this would impact regulated proteolysis. It is well-known that serine proteases of the trypsin type cleave proteins specifically after basic arginine and lysine residues and not after citrulline, as evidenced by specific fluorogenic substrates (Figure 2C). Furthermore, proteolysis by the serine protease neutrophil elastase had previously been shown to induce chromatin decondensation in isolated cell nuclei of different origin (7). However, biochemically, neutrophil elastase shows no arginine or lysine-guided specific proteolysis. It was, therefore, not surprising to observe that elastase was able to mediate chromatin decondensation of isolated nuclei independently of protein citrullination by PAD4 (Figures 2D,E). PAD4 alone was able to induce a minor chromatin decondensation in isolated nuclei but failed to reach the pronounced effect of proteolytic nuclear decondensation guided by neutrophil elastase (Figures 2D,E). Ionomycin has been known to promote the activity of calpain proteases (25). Calpain and peptidyl arginine deiminases share the feature that both need high calcium concentrations to fulfill their enzymatic activity. Supported by recent publications, we argued that calpain proteolysis might be enhanced by protein citrullination in nuclear decondensation assays (20, 26). We, therefore, stimulated isolated nuclei with calpain and PAD4 and a combination of both enzymes. We chose non-granulocytic cell lines in this approach to limit confounding effects of intrinsic PADs and granular proteases. Notably, isolated nuclei resisted calpain treatment and their morphology remained unaltered (Figures 2F,G). As observed before (27), recombinant PAD4 alone was able to induce a mild increase in nuclear area of 20%. However, nuclei enzymatically processed by PAD4 suddenly showed a striking susceptibility to calpain: In response to calpain, the nuclear area strongly expanded and displayed a reduced intensity as observed in NET formation (Figures 2F,G). This effect was partially reversed by EDTA. We next explored the effect of citrullination enhanced calpain-mediated nuclear decondensation in an independent preparation of mouse embryonic fibroblast nuclei. As observed in A549 cell nuclei, combined challenge with both enzymes induced a marked chromatin decondensation in MEF nuclei, whereas single treatment failed to strongly decondense the nuclei (Figure S1). Taken together, proteolysis of nuclear proteins differs in PMA- and ionomycin-induced chromatin decondensation. Citrullination selectively affects proteolytic events. Whereas, elastase can decondense nuclei irrespective of citrullination, PAD4 strongly enhances nuclear decondensation by calpain.

### Breakdown of the Nuclear Envelope and Chromatin-Bound Proteins by the Concerted Action of PAD4 and Calpain-Mediated Proteolysis

**Figure 3:**
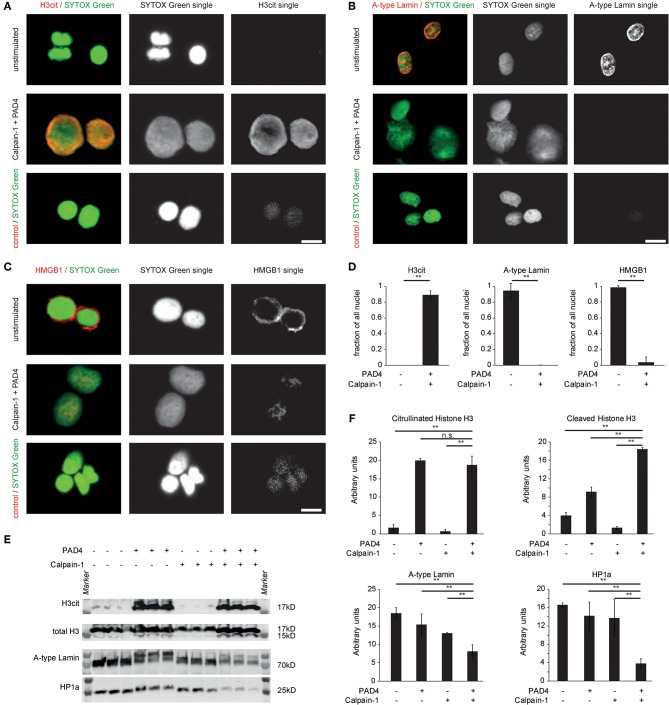
Breakdown of the nuclear envelope and chromatin-bound proteins by the concerted action of PAD4 and calpain-mediated proteolysis. MEF nuclei were subjected to human calpain-1 after preincubation in the presence of PAD4 or left unstimulated and processed for immune cytochemistry of **(A)** citrullinated Histone H3, **(B)** Lamin A+C, and **(C)** HMGB1. DNA counterstaining was performed using SYTOX Green (scale bar = 20 μm) **(D)** H3cit^+^, lamin A+C^+^ and HMGB1^+^, respectively, were quantified as a fraction of all nuclei stained by SYTOX Green (^**^p < 0.01, student's *t*-test); data derived from 3 independent experiments). **(E)** Isolated A549 nuclei were subjected to the enzymatic activity of either calpain-1, PAD4 or the consecutive combination of both enzymes for 60 min each. Subsequently, proteins were isolated and processed for western blotting as indicated. **(F)** Band intensity calculations of western blots as in **(E)** were performed (^**^p < 0.01, ANOVA and *post-hoc* Tukey HSD test). Three independent experiments were performed with similar results.

We next explored the consequences of citrullination and calpain-mediated proteolysis on the protein level. Isolated nuclei were amenable to extensive histone H3 citrullination and subsequent chromatin decondensation when treated with recombinant PAD4 and human calpain (Figure 3A). Nuclear envelope disintegration was observed by immunocytochemistry. Untreated isolated MEF nuclei displayed surrounding lamin A+C positive nuclear lamina. After citrullination and calpain-mediated proteolysis, the nuclear lamina was lost (Figure 3B). Also HMGB1 was detected in the border area of the isolated nuclei (Figure 3C) (its chromatin association was possibly shifted during the isolation protocol). The concerted action of PAD4 and calpain removed the HMGB1 positive border surrounding the nucleus and induced a pronounced and significant nuclear decondensation (Figure 3D). These effects were also assessed by western blot analyses. The previously identified targets of proteolysis in NETs were specifically lost in MEF nuclei treated with both of these enzymes. Citrullination by PAD4 is known to delay migration in gels due to loss of charge leading to two separate bands in the presence of PAD4. Loss of A-type lamins by calpain (Figures 3E,F) was favored in the presence of PAD4. Additionally, the cleavage of histone H3 at its N-terminus as well as of HP1a was observed. Taken together, nuclear decondensation in response to PAD4 and calpain showed a marked citrullination of chromatin-bound histones by PAD4 and induced nuclear decondensation accompanied by a loss of lamins and chromatin-bound proteins, most likely mediated by proteolysis.

### Concerted Action of PAD4 and Calpain Determines Nuclear Envelope Breakdown in Calcium Ionophore-Induced NET Formation

**Figure 4:**
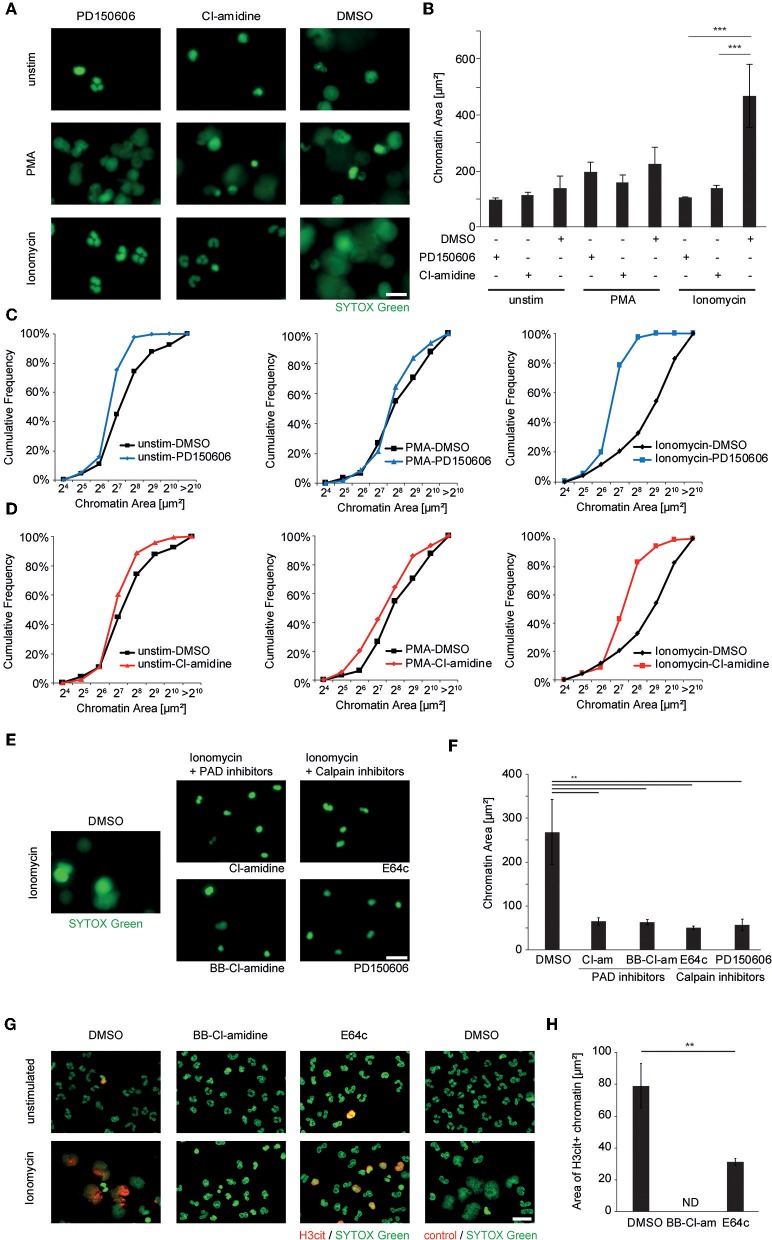
Ionophore-induced chromatin decondensation of human neutrophil granulocytes requires both PAD and calpain activity. Human neutrophils from the peripheral blood of healthy donors were stimulated with PMA (50 nM), Ionomycin (5 μM) or left unstimulated after pre-treatment with DMSO control, the specific calpain inhibitor PD150606 (50 μM) or the PAD-inhibitor Cl-amidine (200 μM), respectively. **(A)** The morphology and spread area of the chromatin was analyzed after 2 h as assessed via fluorescence microscopy after SYTOX Green-mediated DNA staining. Representative images of at least four independent experiments are shown (scale bar = 20 μm). **(B)** Mean + SEM of the median chromatin area of at least 100 single cells of four independent experiments is depicted (^***^p < 0.001, ANOVA and *post-hoc* Tukey HSD test). **(C,D)** Histogram plots showing the distribution of single chromatin configurations as counted in **(B)** after PMA or ionomycin challenge with either PD150606 **(C)** or Cl-amidine **(D)** inhibitor treatment. **(E,F)** The effect of additional inhibitors of PAD enzymes and cysteinyl proteinases on ionomycin-induced chromatin decondensation was assessed using BB-Cl-amidine (20 μM), Cl-amidine (200 μM) and E64c (50 μM) and PD150606 (50 μM). Representative images are shown in **(E)** and chromatin area quantification in **(F)** (scale bar = 20 μm, ^**^p < 0.01 in one-way ANOVA and *post-hoc* Tukey HSD tests, *n* = 3 independent experiments). **(G)** Granulocyte cultures stimulated as indicated were subjected to H3cit immunocytochemistry (scale bar = 20 μm) and **(H)** the area of H3cit immunopositive chromatin after ionomycin stimulation was calculated in each respective condition (^**^p < 0.01 student's *t*-test, *n* = 3 independent experiments; ND, not detected).

In order to further test our hypothesis of synergistic effects of PAD4-mediated citrullination and calpain-directed proteolysis in nuclear decondensation, we assessed the role of calpain and PAD4 in calcium-induced NET formation in human and murine neutrophils. Therefore, we treated healthy-donor-derived peripheral blood neutrophils with PMA and ionomycin as shown before. Pharmacological inhibition of calpain was achieved using PD150606. Inhibition of peptidyl arginine deiminases was achieved using the pan-PAD inhibitor Cl-amidine. Neither calpain inhibition nor Cl-amidine was able to inhibit NET formation induced by PMA, in which PAD activity was not as strongly observed. However, both calpain and PAD inhibition independently inhibited the nuclear decondensation observed in response to ionomycin as depicted by fluorescence microscopy (Figure 4A), nuclear area measurement (Figure 4B) and assessment of the cumulative distribution of the nuclear area (Figures 4C,D). This was further confirmed as ionomycin-induced neutrophil nuclear decondensation was significantly inhibited by two independent calpain inhibitors (PD150606 and the cysteinyl protease inhibitor E64c) and two PAD inhibitors (Cl-amidine, BB-Cl-amidine), respectively (Figures 4E,F). Pharmacological inhibition of PADs blocked ionomycin-inducible histone citrullination and chromatin decondensation in neutrophils. After pharmacological inhibition of calpains, citrullination was still evident in ionomycin-stimulated neutrophils. However, nuclear decondensation was blocked when calpain activity was inhibited (Figures 4G,H).

### PAD4 Is Essential for ionophore-Induced Chromatin Decondensation and Nuclear Protein Degradation in Murine Neutrophils

**Figure 5:**
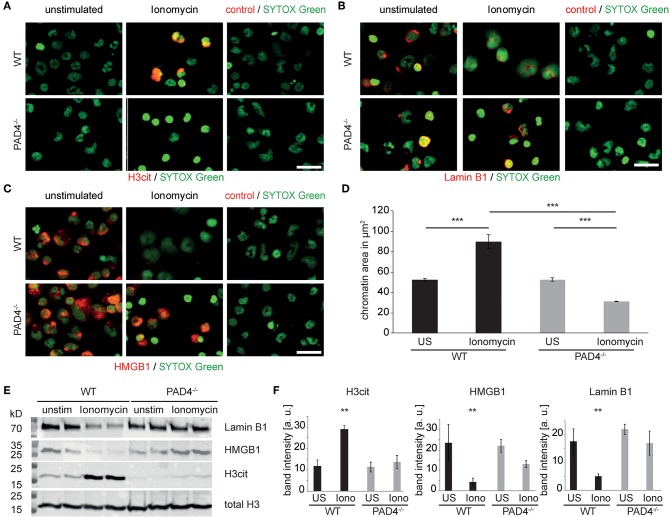
PAD4 is essential for ionophore-induced chromatin decondensation and nuclear protein degradation in murine granulocytes. Murine peritoneal neutrophils were stimulated with ionomycin for 120 min or left unstimulated. **(A)** H3cit immunocytochemistry reveals PAD4 activity in WT cells and its absence in PAD4-deficient neutrophils (*n* = 3 independent experiments). **(B)** Identical cultures were subjected to Lamin-B1 and **(C)** HMGB1 immune cytochemistry. **(D)** The morphology and spread area of the chromatin was analyzed after 2 h as assessed via fluorescence microscopy after SYTOX Green-mediated DNA staining (*n* = 4 independent experiments with at least 100 nuclei per analysis, ^***^p < 0.001, ANOVA and *post-hoc* Tukey HSD test). **(E)** Western blots of total histone H3, citrullinated histone H3, Lamin B1, and HMGB1 of murine peritoneal neutrophil cultures were performed. One representative blot is presented. **(F)** Results of four independent western blots as in **(E)** were used for band intensity quantifications (all scale bars = 20 μm, ^**^p < 0.01 ANOVA and *post-hoc* Tukey HSD).

In order to assess the direct contribution of PAD4 to nuclear proteolysis independently of pharmacological inhibitors, we stimulated murine peritoneal neutrophils with ionomycin and assessed the integrity of the previously identified nuclear targets of proteolysis. Wildtype neutrophils displayed marked increases in histone H3 citrullination in response to ionomycin, which was absent in PAD4-deficient cells (Figure 5A). While ionomycin triggered a marked loss of both lamin B1 (Figure 5B) and HMGB1 (Figure 5C), this did not occur in PAD4-deficient cells. Wild-type neutrophils responded to ionomycin with robust chromatin decondensation (Figure 5D), while PAD4-deficient neutrophils failed to decondense nuclear chromatin in response to ionomycin. Impressively, ionomycin-induced H3 citrullination was associated with Lamin B1 and HMGB1 loss in wild-type neutrophils. However, deficiency of PAD4 not only blocked H3 citrullination but furthermore significantly inhibited lamin B1 and HMGB1 degradation (Figures 5E,F).

### Citrullination by PAD4 Facilitates Proteolysis of HMGB1 by Calpain

**Figure 6:**
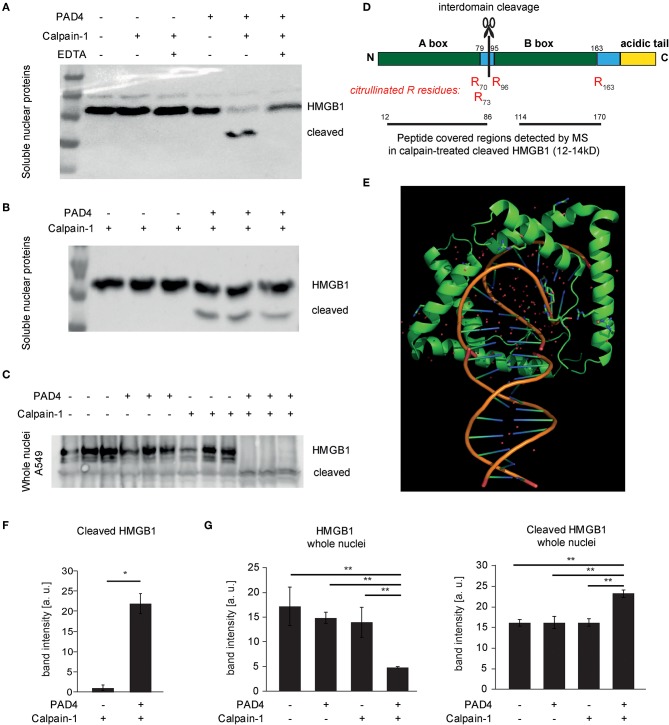
Cleavage of HMGB1 by calpain and its selective dependence on PAD4. **(A,B,F)** Soluble nuclear protein lysates of A549 cells were subjected to PAD4-mediated protein citrullination and consecutive calpain-1-mediated proteolysis. HMGB1 was visualized by western blotting using a C-terminal primary anti-HMGB1 antibody (ab18256) (representative of 3 independent experiments). **(C,G)** Intact isolated A549 nuclei were subjected to enzymatic treatments as in **(B)** and subjected to western blotting. **(D**) The molecular organization of HMGB1 is depicted showing the two DNA-binding domains (Box A, Box B) and the acidic tail. Furthermore, the citrullinated arginine residues detected by mass spectrometry (MS) in cleaved HMGB1 (12–14 kD) are depicted in red (R70, R73, R96, R163). Regions covered by peptides identified in the cleaved HMGB1 fragment by MS are highlighted. **(E)** A structural representation of DNA-binding of two A-boxes of HMGB1 (*in green*) is depicted showing its binding to kinked DNA (based on PDB: 4QR9) (28). **(F)** shows respective relative band intensity quantifications of cleaved HMGB1 (student's *t*-test, ^*^p < 0.05, representative of 3 independent experiments) **(G)** Intact isolated A549 nuclei were subjected to enzymatic treatments as indicated and subjected to western blotting. Relative band intensity quantifications are presented (ANOVA and *post-hoc* Tukey HSD, ^**^p < 0.01, representative of 3 independent experiments).

We further characterized calpain-mediated cleavage and citrullination *in vitro* using subcellular protein fractionation. Both soluble and chromatin-bound nuclear proteins were subjected to citrullination and calpain-mediated proteolysis. These analyses showed PAD-independent proteolysis of large spectrin repeat proteins e.g., Filamin and alpha-actinin as identified by mass spectrometry. However, at 24–27 kD, protein bands were apparent, that selectively disappeared after calpain cleavage and citrullination (Figure S2). HMGB1 was identified as a candidate protein: In soluble nuclear protein lysates of A549 cells, enhanced proteolysis of HMGB1 facilitated by citrullination was apparent (Figures 6A,B,F). Even more pronounced was the joint effect of calpain and PAD4 on proteolysis of HMGB1 in whole isolated nuclei (Figures 6C,G): Here, loss of HMGB1 was nearly complete, probably favored by protein conformation. HMGB1 is characterized by two L-shaped DNA-binding domains allowing the preferential binding of HMGB1 to kinked DNA (Figures 6D,E). Interestingly, calpain-mediated cleavage generated a cleaved form of HMGB1 that was detected in significantly increased amounts after nuclear protein citrullination (Figures 6F,G) using a polyclonal antibody directed against a C-terminal peptide of human HMGB1. In line with the concept of interdomain cleavage, the fragment was approximately half of the full-length HMGB1 (12-14kD). Recombinant HMGB1 was treated with PAD4 and Calpain-1 and the cleavage fragments were analyzed by mass-spectrometry (Figure 6D). Interestingly, HMGB1 showed evidence of citrullination specifically at the edges of the A- and B-box (R_70_, R_73_, R_96_, R_163_). Peptides spanning both A-box and B-box were detected in the cleaved band. This argues for an interdomain cleavage resulting in two fragments of similar size. We speculate that citrullination at the edges of the boxes facilitated calpain-mediated cleavage.

Taken together, we propose a central role for peptidyl arginine deiminase 4 in the regulation of calpain-mediated proteolysis of proteins of the nuclear lamina and core, as exemplified by lamins and HMGB1. The concerted action of calpain and PAD4 contributes to nuclear decondensation as observed in ionophore-induced NET formation.

## Discussion

In recent years substantial progress has been made in deciphering the functional role of NETs *in vivo*. Yet, seemingly contradictory findings regarding the molecular mechanisms of NET formation have contributed to confusion in a quickly expanding field of research (7, 9, 10, 12, 29). The landmark publications nicely described NET formation in response to phorbol-myristate acetate (PMA) and the necessity of the oxidative burst as a paradigm of NET formation (1, 3). PMA-induced NET formation further depends on the PI3K/Akt and the Ras/RAF/MEK/ERK pathway (30). Some of these features are also evident in response to microbial stimuli (31). On the other hand, NET formation has been described in response to calcium ionophores such as ionomycin (9, 12, 22, 32). Calcium-induced NET formation may occur in response to integrin-mediated NET formation (33, 34) or stimuli such as purins (35), pyroptotic membrane pores (36), bicarbonate-pCO2 alterations (29, 37) or calcium store-operated mechanisms (38). Calcium ionophore-induced NET formation is largely independent of NADPH oxidase, whereas mitochondrial ROS may play an important role (39, 40). Calcium ionophore-induced NET formation shows a striking dependency of peptidyl arginine deiminases as chromatin decondensation is blocked in the presence of both pan-PAD and selective inhibitors of PAD4 (41). Taken together, PMA and ionomycin induce independent pathways resulting in the extrusion of decondensed chromatin. As previously observed citrullination is rather restricted to calcium-dependent NET formation (32). Citrullination may also occur in neutrophils after PMA stimulation, once cell lysis has occurred, but does not seem to contribute to earlier chromatin decondensation (11). Furthermore, neutrophil chromatin decondensation in response to calcium ionophores is blocked in PAD4-deficient mice (10). In agreement with the concept of the existence of independent pathways of chromatin decondensation in neutrophils, Holmes et al. observed that decondensed chromatin, was still observable after ionomycin treatment of PAD4-deficient neutrophils, though strongly less abundant than in wild-type counterparts, most probably derived from citrullination-independent pathways (42).

PAD enzymes specifically citrullinate arginine residues. This property can strongly alter the susceptibility of proteins to proteolysis: Serine proteases with tryptic activity cleave behind basic amino acids such as arginine and lysine and citrullination inhibits trypsin-mediated cleavage after modified residues (43). The trypsin-related neutrophil serine protease Cathepsin G failed to induce chromatin decondensation in previous studies (7). It has been recently proposed, that citrullination actually increases serine protease activity by inhibiting serpin protease inhibitors (44). Many serpins make use of arginine baits in the P1 position to inhibit the target serine protease. Citrullination alters the bait and effectively blocks serpin inhibitory activity. Tilvawala et al. propose the complement system to have an important role in cell lysis. The experiments presented in our work were performed under serum-free conditions, making an important contribution of the complement system to our observations unlikely. Neutrophil elastase, previously implicated in PMA-induced NET formation, does not display arginine preference and is inhibited by serpins that do not contain an arginine at the P1 position (7). Proteolytic activity of neutrophil elastase, thus, appears unrelated to direct effects of PAD4, as shown above.

For many years, calpain has been implicated in cell death routines induced by high levels of intracellular calcium, especially in the setting of neurodegenerative diseases (45). Calpains are best known for limited interdomain cleavage of their many substrates which include cytoskeletal repeat domain proteins e.g., spectrins and mitochondrial factors, such as apoptosis inducing factor AIF, Bax, Bid or Bcl-2 (46–48). While it is known that nuclear proteins can be targets of calpain-induced proteolysis, little is known about the nuclear role of calpains (49). Inspired by the fact that peptidyl arginine deiminases and calpain share the requirement of high calcium concentrations and independent studies, which have shown facilitated calpain cleavage of citrullinated proteins (20, 26), we hypothesized that calpain and PAD4 synergize in ionophore-induced chromatin decondensation and NET formation. Activation of many membrane-bound receptors may lead to increased cytosolic calcium levels. Ionophore treatment bypasses specific receptor engagement, yet is a valid tool in assessing the downstream consequences of marked increases of cytosolic calcium levels.

Indeed, we observed, that calpain-1 alone did not induce chromatin decondensation. PAD4-treatment only modestly relaxed chromatin. However, incubation of citrullinated nuclei with calpain induces a marked and robust chromatin decondensation almost to the magnitude observed by elastase treatment. In an unbiased approach, we aimed to identify targets of calpain-1, targets of citrullination and proteins that were selectively cleaved by calpain-1 in the citrullinated state. These experiments revealed that large multi-repeat domain proteins in nuclear protein lysates (e.g., α-Actinin-4 and Filamin A as identified by mass spectrometry), once accessible, do not necessarily require citrullination in order to be efficiently processed by calpain-1. On the other hand, this approach revealed a band of 24–27 kD, which was selectively processed after citrullination of nuclear proteins (Figure S2). Western blot analyses of selected proteins identified HMGB1 as a protein of interest. Taken together, we observed a crucial synergy of calpain and PAD4, by which PAD4 licenses calpain to decondense chromatin.

How do calpain and PAD4 synergize in nuclear lysis and chromatin decondensation on a molecular level? To address this, we treated recombinant HMGB1 with calpain and PAD4. Citrullination of R residues was detected specifically at the edges of the DNA-binding A- and B-boxes. Tarcsa et al. described that citrullination favorably occurs in disordered regions of a protein (50). In line with this, citrullination at the edges of the domains and consecutive loss of polar interactions in this critical area might facilitate inter-domain cleavage by calpain. In line with substrate citrullination, citrullinated lamin C has been previously described (51). Thus, our experimental evidence points toward a synergy of calpain and PAD4 on the substrate level.

Additional modes of synergy of calpain and PAD4 might exist. Konig et al. detected calpastatin, the natural inhibitor of calpain among citrullinated proteins of human neutrophils (23). We speculate that citrullination might affect the interaction of calpain with its natural inhibitor. In the *in vitro* assays presented here, however, calpastatin was not present.

**Figure 7:**
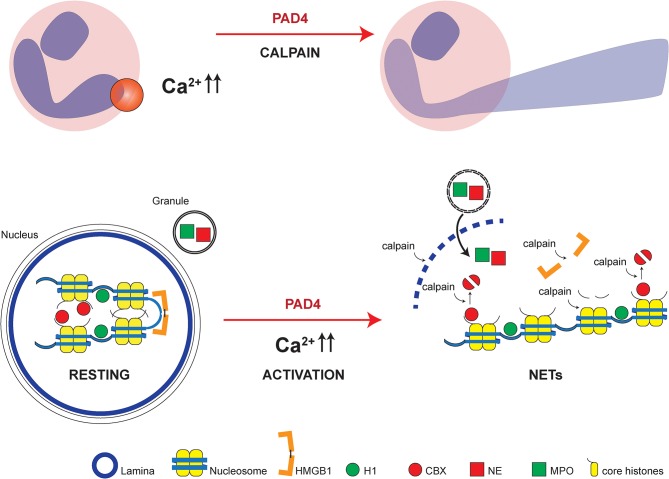
Model of NET formation dependent on calpain and PAD4. Localized PAD and calpain activity allows local nuclear lamina breakdown and chromatin decondensation leading to disturbed polar protein interactions due to citrullination and consecutively increased accessibility and facilitated proteolytic cleavage by calpain of protein substrates. Cytosol-wide calcium increases in response to calcium ionophores induce wide-spread chromatin decondensation reminiscent of NET formation. High mobility group box protein 1 (HMGB1), histone H1 (H1), chromobox heterochromatin protein (CBX), neutrophil elastase (NE) and myeloperoxidase (MPO), and core histones with N-termini are highlighted.

Our study integrates regulated proteolysis by calpain to the pathways of NET formation. Citrullination of chromatin and loss of charge of chromatin-associated proteins, as proposed by Wang et al. (12) may still be an important driving force in chromatin decondensation. However, we propose that efficient nuclear lysis requires additional proteolysis of the nuclear lamina and chromatin-bound proteins. These observations are also relevant to integrate seemingly contradictory observations regarding the studies of “vital” NET formation: Independent labs observed that NET formation *in vitro* is mostly associated to cell death but it was also discovered that under certain conditions nuclear expulsion occurs *in vivo* with remaining “viable” anuclear cytoplasts which still migrate and potentially phagocytize (52, 53). Calpain activity directly cleaves cytoskeletal components in a regulated fashion and is known for its essential functions both in the course of cell death and migration of viable cells (54–56). We speculate that disintegrating the cytoskeleton in a calpain- and PAD4-dependent manner on a specific location of the cytoskeleton can leave the rest of the cell body intact, while allowing targeted NET release possibly in response to receptor or adhesion molecule signaling (Figure 7). Future studies will need to provide further experimental evidence for this concept.

Taken together, we show that PAD4-mediated citrullination is a crucial prerequisite of nuclear decondensation in ionophore-induced NETosis coinciding and synergizing with specific calpain-directed cleavage of the nuclear lamina and chromatin-bound proteins. This finding links PAD4-mediated protein citrullination to calpain-directed proteolysis, considers calpain in the pathways of NET formation and thus proposes a novel mechanistic role of PAD4 in NET formation.

## Materials and Methods

### Antibodies

**Table:** 

**Primary antibodies**	**Source**	**Dilution [ICC, WB]**
ATP1A1	RRID:AB_2060983	WB 1:1000
Citrullinated Histone H3 (H3cit)	RRID:AB_304752	[1:200, 1:1000]
Neutrophil Elastase	RRID:AB_446409	[1:200, 1:1000]
GAPDH	RRID:AB_10622025	WB 1:1000
High Mobility Group Box Protein 1 (HMGB1)	RRID:AB_1139336	[1:200, 1:1000]
HP1 alpha	RRID:AB_10858495	[1:100, 1:1000]
Lamin A/C	RRID:AB_10860619	[1:500, 1:1000]
Lamin B1	RRID:AB_10107828	[1:200, 1:1000]
Modified citrulline	RRID:AB_10097769	According to recommended protocol
PARP	RRID:AB_777101	WB 1:1000
Vimentin	RRID:AB_10695459	WB 1:1000

### Mice

PAD4^−/−^ were kindly provided by K. Mowen, Scripps Institute, La Jolla, CA, USA and have been described previously (8). All mice studied were on the C57Bl/6 background. Mice aged 6–14 weeks were used for experimental procedures. All mice were kept under specific pathogen-free conditions at the animal facility of the University of Erlangen. Experimental procedures were approved by the local committees of Lower Franconia.

### Cell Isolation Procedures

Human peripheral blood neutrophils were isolated from healthy donors and a patient suffering from CGD (hypomorphic gp91 mutation) after informed consent in agreement to local ethical regulations (Application No. 4489) and separated using PanColl (PanBiotech, Germany) density gradient centrifugation. Granulocytes were enriched from the erythrocyte pellet by consequent dextrane sedimentation (60 min, 1%, Carl Roth, Germany). The purity of neutrophil isolations was routinely above 90%. Murine neutrophils were isolated from the peritoneal cavity by peritoneal lavage of HBSS-EDTA solution and subsequent centrifugation on day 1 after thioglycolate instillation (3%) to the peritoneal cavity. Cells were counted and directly recultured in HBSS-based media (HBSS, 2 mM CaCl_2_, 4 mM NaHCO_3_) for further stimulation.

### Neutrophil Stimulations and Treatment With Pharmacological Inhibitors

Neutrophil suspensions of human and murine origin were cultured in Hank's buffered saline solution containing 4 mM NaHCO_3_ and 2 mM CaCl_2_ and stimulated with the biochemical stimuli phorbol myristate acetate (Cayman Chem, 50 nM) and ionomycin (Cayman Chem, 5 μM) in the presence or absence of the peptidylarginine deiminase inhibitors Cl-amidine (Merck Millipore, 200 μM), BB-Cl-amidine (Cayman Chem, 20 μM), and the cysteinyl protease inhibitors E64c (Santa Cruz, 50 μM) and PD150606 (Santa Cruz, 50 μM). Pharmacological inhibitors were allowed to preincubate in the cell culture at 37°C/5%CO_2_ for 1 h before stimulation. Neutrophil nuclear morphology was assessed directly after 120–180 min of cell culture via SYTOX Green-mediated chromatin staining and fluorescence microscopy.

### Confocal Laser-Scanning Microscopic Analysis of Neutrophils

Neutrophil granulocytes were isolated, stimulated and consecutively processed for immune cytochemistry as indicated above. Stained glass slides (ibidi, BD Biosciences) were subjected to confocal laser-scanning microscopy (Leica inverted SP5) and Z-Stacks were acquired. Maximum intensity projections of Z-stacks (4.5, 0.5 μm slices) are depicted. Further image processing was achieved by Leica, Image J and Adobe Photoshop software.

### Determination of Chromatin Decondensation

Size and area calculations of enzymatically treated nuclei and neutrophils were performed using fluorescent images of SYTOX Green-stained cell and nuclear cultures assisted by Adobe Photoshop measurement tools. Mean, median and standard deviation were calculated using Microsoft Excel. Mean of the median of single independent experiments and the standard error was calculated and presented. In additional approaches neutrophil nuclear morphology was determined from confocal laser-scanning microscopic pictures after immune cytochemistry.

### Platereader-Based Quantification of Fluorescence

Plates containing polymorphonuclear granulocyte (PMN) cultures were analyzed under the conditions described above for 180 min in an infinite^®^ 200 plate reader (TECAN, Germany). Excitation was performed at 488 nm and emission was detected at 523 nm. ROS were detected using H2-DCFDA and neutrophil chromatin was detected using SYTOX Green (ThermoFisher Scientific). Trypsin-mediated cleavage of H-Cit-AMC and H-Arg-AMC (25 μM, Bachem, Sigma Aldrich) was assessed in trypsin activity buffer (67 mM Sodium Phosphate Buffer, pH 7.6 at 25°C).

### Immunocytochemistry

Immunofluorescence of cells stimulated in BD Culture Slides was performed as described below and recorded on either a confocal laser scanning-microscope or a standard fluorescence microscope (Leica, Germany) using overnight hybridization with primary Abs specific for the target proteins. Concentrations and antibody sources can be deduced from the table above. Detection was performed using directly labeled Alexa 488 or Alexa 555-conjugated target species antibodies (Abcam, 1:200–1:1000). Before examination, the nuclei were counterstained with either SYTOX Green or NucRed (Invitrogen Molecular Probes, Karlsruhe, Germany; BD, Heidelberg, Germany).

### Nucleus Isolation Protocol

A549 cells and mouse embryonic fibroblasts were grown in T75 culture flasks in DMEM media, pelleted and washed in NB nucleus isolation buffer [10 mM HEPES pH 7.4, 10 mM KCl, 2 mM MgCl2, 1 mM DTT, PMSF, phosphoSTOP (Roche)]. Cells were lysed in NB+0.2% Triton-X for 30 min with intermittent vortexing. Lysed cells were washed in NB buffer and subjected to a sucrose gradient (NB buffer + 30% sucrose, 10′ centrifugation at 1,300 rpm, 4°C) and washed. A sample of the isolated nuclei was counterstained with Hoechst and the yield was microscopically counted. Isolated nuclei were enzymatically processed as indicated below or transferred to storage buffer (50 mM Tris-HCl pH 7.5, 250 mM Sucrose, 25 mM KCl, 5 mM MgCl_2_, 1 mM DTT) for cryoconservation and subsequent protein isolation procedures.

### Enzymatic Treatment of Proteins and Nuclei

Isolated nuclei, recombinant histones and nuclear protein fractions were enzymatically treated with recombinant PAD4 (Cayman Chem, 5 μM) for 90 min in PAD activity buffer (100 mM Tris-HCl, 10 mM CaCl_2_, 5 mM DTT). Consecutive incubation in the presence of human calpain-1 (Sigma) was performed for 4–16 h without a change in buffer conditions. For blotting and detection of citrullination and cleavage, recombinant proteins (2 μg per lane) and nuclear protein lysates (15 μg per lane) were consecutively treated with PAD4 (112 ng/624 ng) and human calpain-1 (400 ng/1 μg). Nuclei were digested by neutrophil elastase (60 nM, Sigma) in 100 mM Tris-HCl, 10 mM CaCl_2_, 5 mM DTT after treatment with or without recombinant PAD4 (5 μM).

### Protein Isolation Techniques

Subcellular protein fractions were isolated using a ready-made kit following the instructions by the manufacturer (ThermoFisher Scientific). Granulocyte proteins and proteins of enzymatically treated isolated nuclei were isolated after adding HALT protease inhibitor (ThermoFisher Scientific) directly to the culture well and subsequent prompt heat denaturation at 95°C for 5 min in LDS gel loading buffer. Protein quantification was performed using Bradford reagent (Carl Roth). For further analysis, Western blots were performed after SDS-PAGE using ready-made gels (Bio-Rad). Digital image acquisition was performed using a Bio-Rad ChemiDoc Imaging System (Bio-Rad). Images of molecular size markers and specific Western Blots were merged using the manufacturer's recommended software. Nitrocellulose membranes of western blots were reprobed after washing in Tris buffered saline, 0.1% Tween-20 (TBS/T) and stripping in Tris-HCl pH 6.8, SDS and β-mercaptoethanol.

### Mass Spectrometric Protein Identification

Bands from Coomassie-stained gels were excised using a sterile scalpel. The proteins were identified following trypsin in-gel digestion by matrix-assisted laser desorption ionization (MALDI)–time of flight (TOF) MS via peptide mass fingerprinting using a Bruker Ultraflex TOF/TOF MALDI instrument (Bruker Daltonics, Bremen, Germany). The instrument was operated in the positive-ion reflectron mode using 2,5-dihydroxybenzoic acid and methylenediphosphonic acid as a matrix. Sum spectra consisting of 200–400 single spectra were acquired. For data processing and instrument control, the Compass (version 1.1) software package, consisting of FlexControl (version 2.4), FlexAnalysis (version 3.0), and BioTools (version 3.0), was used. The proteins were identified via a MASCOT peptide mass fingerprint search (Matrix Science) using the NCBInr database.

### Statistical Analysis

Data were analyzed as indicated using the unpaired Student's *t*-test using Microsoft Excel (Microsoft, Redmond, WA) or an analysis of variance (ANOVA) with *post hoc* Tukey HSD tests as indicated. When appropriate, correction for multiple testing was performed. Figure preparation was performed using Adobe Creative Suite CS5/CS6, PyMOL and the Microsoft Office Suite 2010.

## Data Availability Statement

All datasets generated for this study are included in the article/Supplementary Material.

## Ethics Statement

The studies involving human participants were reviewed and approved by Ethikkommission UK Erlangen. The patients/participants provided their written informed consent to participate in this study. The animal study was reviewed and approved by Regierungsbezirk Unterfranken, Germany.

## Author Contributions

ML conceived the study and designed the experiments. SG, AL, CM, and ML performed the experiments. ML drafted the manuscript. PG, MB, MH, and NN-B provided valuable experimental tools and advice for this study, mass spectrometry was performed and evaluated by GL. All authors edited the manuscript.

### Conflict of Interest

The authors declare that the research was conducted in the absence of any commercial or financial relationships that could be construed as a potential conflict of interest.

## Supplementary Material

Figure S1 PAD4 prepares nuclei for calpain-mediated proteolysis. **(A)** Isolated MEF nuclei were subjected to human calpain-1 (1 μg) for 16 h after preincubation in the presence or absence of PAD4 (5 μM) for 90 min. **(B)** Nuclear chromatin area of SYTOX-Green-stained single nuclei was quantified (at least 100 nuclei per one of 3 independent experiments, depicted as mean + SEM, scale bar = 20 μm), ^***^*p* < 0.001, ANOVA and *post-hoc* Tukey HSD test).

Figure S2 Subcellular fractionation and protein isolation for subsequent enzyme treatment. **(A)** A549 cells were subjected to subcellular fractionation and protein isolation according to manufacturer's instructions using the respective Thermo Scientific Kit. **(B)** Purity of the fractionation was assessed using Western Blotting of compartment-specific proteins (GAPDH, ATP1A1, PARP, HISTH3, VIM). **(C)** Isolated A549 nuclear proteins were subjected to the enzymatic activity of either calpain-1, PAD4 or the consecutive combination of both enzymes (PAD4 90 min, calpain-1 90 min). Selective bands were analyzed using LC/MS. The proteins in the indicated bands in lanes 1, 3, 4, and 6 were identified as Filamin A (Mascot-Score 211-306) and alpha-Actinin 4 (Mascot-Score 131-163).
