# Supplementary figures and images for: Citrullination Licenses Calpain to Decondense Nuclei in Neutrophil Extracellular Trap Formation
Source: Front Immunol. 2019 Oct 22;10:2481. doi: 10.3389/fimmu.2019.02481 (PMC6817590; doi:10.3389/fimmu.2019.02481)

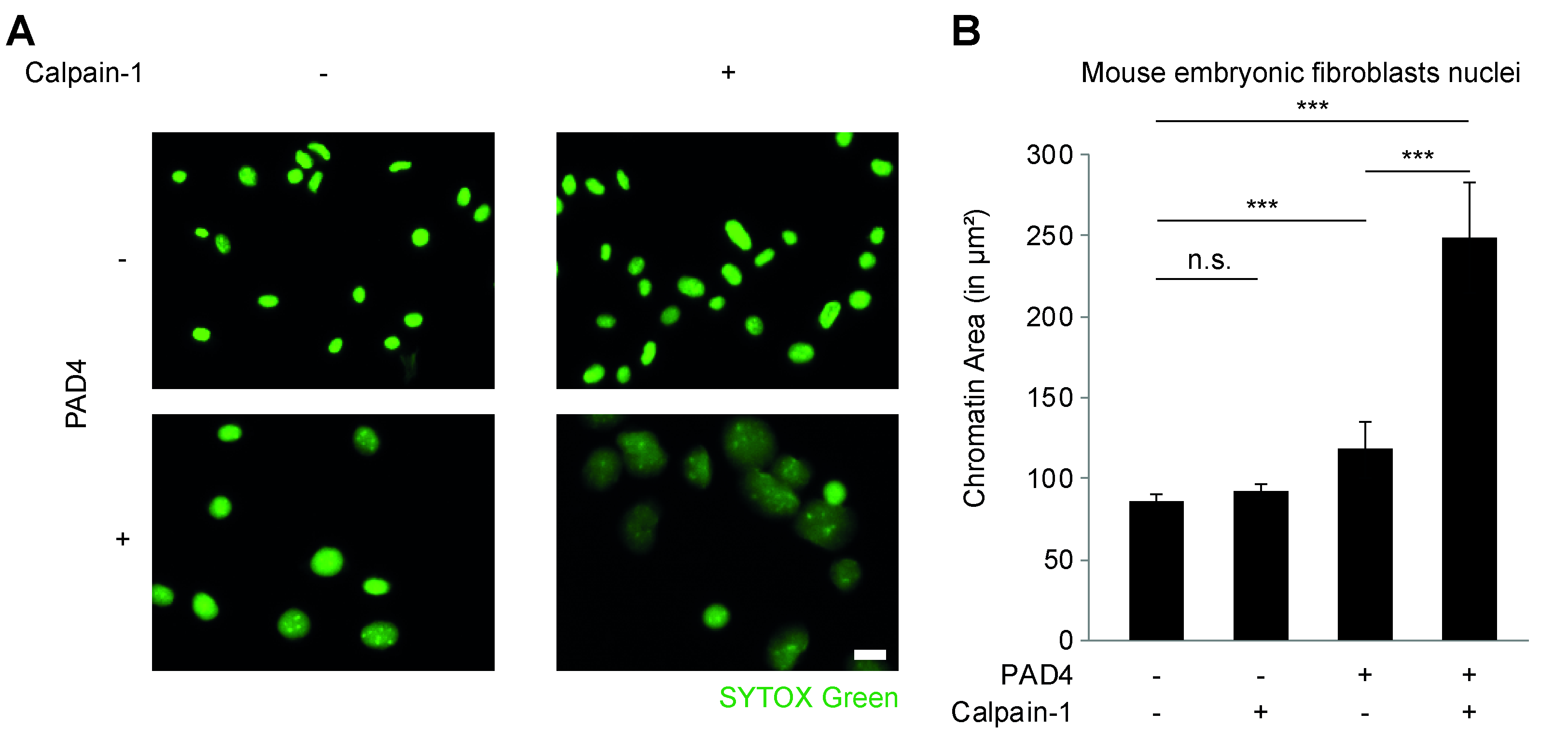

Supplement: Figure S1 — PAD4 prepares nuclei for calpain-mediated proteolysis. (A) Isolated MEF nuclei were subjected to human calpain-1 (1 μg) for 16 h after preincubation in the presence or absence of PAD4 (5 μM) for 90 min. (B) Nuclear chromatin area of SYTOX-Green-stained single nuclei was quantified (at least 100 nuclei per one of 3 independent experiments, depicted as mean + SEM, scale bar = 20 μm), ***p < 0.001, ANOVA and post-hoc Tukey HSD test). [file Image_1.TIF]

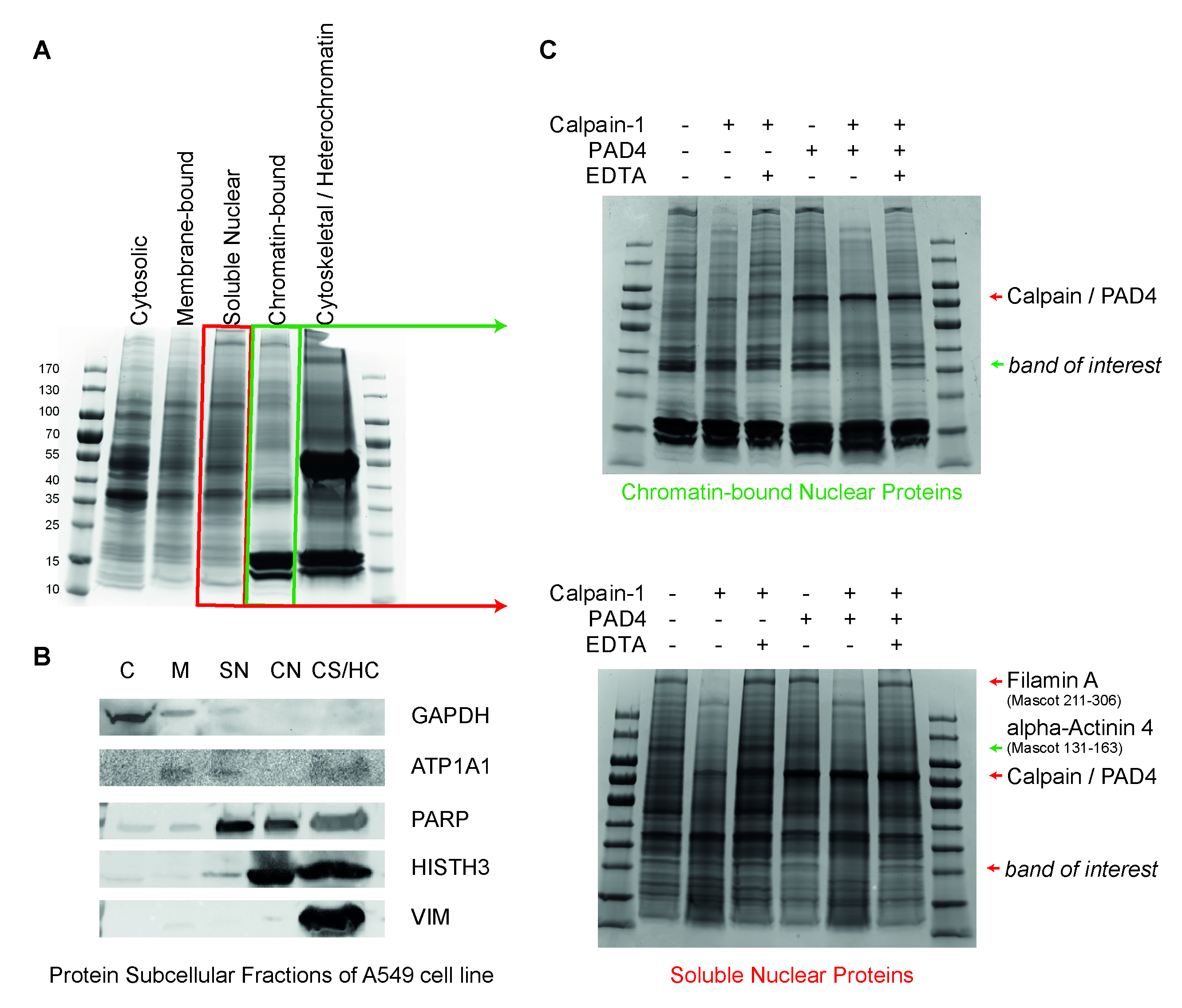

Supplement: Figure S2 — Subcellular fractionation and protein isolation for subsequent enzyme treatment. (A) A549 cells were subjected to subcellular fractionation and protein isolation according to manufacturer's instructions using the respective Thermo Scientific Kit. (B) Purity of the fractionation was assessed using Western Blotting of compartment-specific proteins (GAPDH, ATP1A1, PARP, HISTH3, VIM). (C) Isolated A549 nuclear proteins were subjected to the enzymatic activity of either calpain-1, PAD4 or the consecutive combination of both enzymes (PAD4 90 min, calpain-1 90 min). Selective bands were analyzed using LC/MS. The proteins in the indicated bands in lanes 1, 3, 4, and 6 were identified as Filamin A (Mascot-Score 211-306) and alpha-Actinin 4 (Mascot-Score 131-163). [file Image_2.TIF]
